# Third-Hand Exposure to E-Cigarette Vapour Induces Pulmonary Effects in Mice

**DOI:** 10.3390/toxics11090749

**Published:** 2023-09-04

**Authors:** Andrew E. Thorpe, Chantal Donovan, Richard Y. Kim, Howard J. Vindin, Razia Zakarya, Hanna Miyai, Yik L. Chan, David van Reyk, Hui Chen, Brian G. Oliver

**Affiliations:** 1 School of Life Sciences, Faculty of Science, University of Technology Sydney, Sydney, NSW 2007, Australiayik.chan@uts.edu.au (Y.L.C.); hui.chen-1@uts.edu.au (H.C.);; 2 Respiratory Cellular and Molecular Biology, Woolcock Institute of Medical Research, Macquarie University, Glebe, NSW 2037, Australia; 3 Immune Health Program, Hunter Medical Research Institute, University of Newcastle, Newcastle, NSW 2000, Australia; 4 School of Life and Environmental Sciences, Faculty of Science, Charles Perkins Centre, The University of Sydney, Sydney, NSW 2006, Australia; 5 Epigenetics of Chronic Disease, Woolcock Institute of Medical Research, Macquarie University, Glebe, NSW 2037, Australia

**Keywords:** e-cigarettes, vaping, e-vaping, nicotine, lung function, fibrosis, remodelling

## Abstract

In the last decade, e-cigarette usage has increased, with an estimated 82 million e-cigarette users globally. This is, in part, due to the common opinion that they are “healthier” than tobacco cigarettes or simply “water vapour”. Third-hand e-vapour exposure is the chemical residue left behind from e-cigarette aerosols, which is of concern due to its invisible nature, especially among young children. However, there is limited information surrounding third-hand e-vapour exposure. This study aimed to investigate the pulmonary effects of sub-chronic third-hand e-vapour exposure in a murine model. BALB/c mice (4 weeks of age) were exposed to a towel containing nicotine free (0 mg) e-vapour, nicotine (18 mg) e-vapour, or no e-vapour (sham) and replaced daily for 4 weeks. At the endpoint, lung function was assessed, and bronchoalveolar lavage fluid and lungs were collected to measure inflammation and fibrosis. Mice exposed to third-hand e-vapour without nicotine had alveolar enlargement compared to sham exposed controls. Mice exposed to third-hand e-vapour with nicotine had reduced bronchial responsiveness to provocation, increased epithelial thickening in large airways, increased epithelial layers in small airways, alveolar enlargement, and increased small airway collagen deposition, compared to sham exposed controls. In conclusion, our study shows that third-hand e-vapour exposure, particularly in the presence of nicotine, negatively affects the lung health of mice and highlights the need for greater public awareness surrounding the dangers of third-hand exposure to e-cigarette vapour.

## 1. Introduction

Since the introduction of e-cigarettes in the last decade, vaping has become an epidemic, where global e-cigarette usage has drastically increased from 7 million users in 2011 to 82 million in 2021 [1,2]. E-cigarette products originally gained a large amount of interest as an aid in smoking cessation; however, e-cigarettes are now commonly used for recreational purposes and popular amongst young populations who have never smoked tobacco [3], and the number of e-cigarette users continues to increase [4].

Currently, there is a debate around the safety of e-cigarettes. On the one hand, there is good evidence of the harms of e-cigarettes. For example, a recent (2022) global systematic review demonstrated negative health effects, including unintentional poisoning, acute poisoning through inhalation, seizures, nausea, and throat irritation [5]. In extreme cases, e-cigarette use is associated with significant negative health impacts, including e-vaping-associated lung injury (EVALI) [6], which is strongly associated with the addition of THC to e-cigarettes, but this is not exclusive to THC use. Data from the USA including 2668 hospitalised EVALI cases found that 14% of patients reported exclusive use of nicotine e-cigarettes [7]. Overall, the use of e-cigarettes in never-smokers is generally accepted as negative. However, there is a counter argument, based around the use of e-cigarettes in smokers and patients with smoking-indued diseases, such as COPD. These likely stem from the initial report from Public Health England, which stated that e-cigarettes are 95% safer than cigarettes [8]. Some studies have found reduced symptoms in patients with COPD [9], and some studies have demonstrated the efficacy of e-cigarettes as a quit aid (e.g., 1 year outcomes e-cigarette versus other forms of nicotine replacement were 18% versus 9.9% with 80% in the e-cigarette group still using e-cigarettes and 9% still using nicotine replacement products) [10].

Lung biopsies from patients with 3-8 years of e-cigarette usage displayed histopathology consistent with small airway centred fibrosis [11], and in vivo murine experiments have highlighted that lung pathology has enhanced fibrotic responses after exposure to e-cigarettes [12]. Furthermore, e-cigarette usage increases pro-inflammatory cytokines and chemokines, and reactive oxygen species [13] that can alter mucociliary clearance by increasing Muc5ac levels, cause paralysis of cilia, reduce cilia in a nicotine-dependent manner, and reduce ciliary beat, which impacts the hydration of airway secretions and mucus viscosity [14,15].

The development of fibrosis is typically considered an event secondary to inflammation; however, the development of lung fibrosis may also occur independent of inflammation. For example, studies have shown that inflammation and fibrosis are likely to both develop in the paediatric and neonatal lung independent of one another. Furthermore, in vitro studies have clearly demonstrated that extracellular matrix (ECM) production and deposition occur in mesenchymal cells independent of any immune involvement. In vitro studies from our own group have pioneered much of this research, for example, showing that both bushfire smoke and cigarette smoke induce ECM deposition [16,17,18]. In the context of e-cigarette research, the latter findings have a similarity in that both could be considered to be stimuli derived from the same source (i.e., plant material), but one contains nicotine and the other does not, much like e-cigarettes are available with and without nicotine. The role of nicotine in the pulmonary system is difficult to untangle since many of the pioneering studies often refer to the effects of cigarettes as a nicotine effect, and the findings from these studies are now considered dogma. Furthermore, when studies have explored the effects of nicotine as an isolated chemical, they often use concentrations that are supramaximal. Nonetheless, it is important to understand the effects that nicotine has on cells within the lung. Nicotine binds to the nicotinic acetylcholine receptors; these comprise a complex family of receptors that consist of different subunits and have different intercellular effects. Furthermore, these effects are cell-type specific. In the context of this study, it is important to emphasise that nicotinic acetylcholine receptors are expressed on airway smooth muscle cells [19] and that contractile agonists such as acetylcholine and carbachol are direct agonists of these receptors [20]. Furthermore, there are several studies demonstrating that nicotinic agonists have smooth-muscle-relaxing properties [21,22,23]. Nicotine also has well-documented anti-inflammatory effects [24], and as such, airway smooth muscle contractility may be indirectly modulated through the reduction of procontractile factors from the immune system. In considering the effects of nicotine, it is important to also consider the physiological effects of cotinine, the major nicotine metabolite in the body. There is now considerable evidence, mostly from the brain, that cotinine functions through non-nicotinic acetylcholine receptors [25].

While the harmful impacts of firsthand e-cigarette usage are well-documented, the effects of third-hand exposure are not fully understood. Third-hand exposure is the inadvertent exposure to residue from an aerosol produced by an e-cigarette device. The residue can be found on various surfaces and can persist even after a vaping event has occurred, including fabrics such as clothing, couches and curtains, surfaces such as walls and floors, and human contact via skin and hair [26,27]. Children and infants are most susceptible to the effects of third-hand exposure due to their thinner skin and age-specific behaviours, such as crawling on surfaces and placing objects in their mouth, increasing their sources of exposure [28,29]. Even at low concentrations, the components in the aerosol can affect lung development, promote lung damage, decrease lung function, and cause vulnerability to additional lung insult [14,30].

To date, studies on third-hand e-cigarette exposure are limited. We previously showed that nicotine and propylene glycol are detected in third-hand e-cigarette residues [31], and mice exposed to short-term third-hand e-vapour had significantly reduced inflammatory cells and cytokines, as well as a reduction in lung function compared to sham-exposed mice [31]. However, the sub-chronic effects remain unclear. We hypothesised that sub-chronic third-hand e-vapour exposure at a young age would result in changes in lung function and morphology. Therefore, using a mouse model, we aimed to measure the changes in lung function and structure, as well as inflammatory and fibrotic markers, after sub-chronic exposure to third-hand e-cigarette exposure.

## 2. Materials and Methods

### 2.1. Modelling Third-Hand E-Vapour Exposure

The animal experiments were approved by the University of Technology Sydney Animal Care and Ethics Committee (ETH18-2890). The third-hand e-vapour exposure protocol was performed as previously described [31]. Briefly, male BALB/c mice (4 weeks of age) were housed in a 12 h light/dark system (room temperature of 20 °C ± 2 °C) with water and standard rodent chow provided ad libitum. Mice were randomly divided into three experimental groups, control (sham), 0 mg (no nicotine) e-vapour, and 18 mg (nicotine) e-vapour (n = 10/group). Autoclaved towels (15 × 10 cm/piece, 100% cotton) were exposed to heated tobacco-flavoured e-cigarette vapour (50% propylene glycol/50% vegetable glycerine; Vamper Empire, VIC) with (18 mg) or without (0 mg) nicotine, generated by an e-cigarette device (30 W, 0.5 Ω, 20 puffs, Kangertech NEBOX, Shenzhen, China). Each towel was used only once, and towels with a surface area of 150 cm^2^ were exposed to 20 consecutive puffs (30 W, 0.5 Ω) of e-cigarette aerosol (a total potential exposure or loading of 250 mL of e-cigarette aerosol/towel over a 2 h period. A single towel (15 × 10 cm) was placed in each cage daily. The control group was supplied with a clean towel of the same size and changed daily.

### 2.2. Lung Function and Bronchoalveolar Lavage

Mice were subjected to lung function tests under deep anaesthesia (15 mg/kg xylazine and 90 mg/kg ketamine) using the FlexiVent System (SCIREQ, Montréal, QC, Canada) as previously described [32]. Lung function data were collected following methacholine provocation to measure airway reactivity. Following euthanasia, bronchoalveolar lavage fluid was collected, processed, and stained with May-Grunwald (Sigma-Aldrich, St. Louis, MO, USA) and Giemsa (Sigma-Aldrich, St. Louis, MO, USA) solutions as previously described [32]. Left lung lobes were then fixed in 10% neutral buffered formalin solution for histological analysis. The right lung lobes were snap-frozen in liquid nitrogen and stored at −80 °C for gene expression analysis.

### 2.3. Histology and Immunohistochemistry

Left lung lobes were processed as previously described [32]. Briefly, lungs were inflated and fixed using a set volume of 500 μL of formalin, processed using the Epredia Excelsior^TM^ AS Tissue Processor (Thermo Scientific, Waltham, MA, USA), paraffin-embedded, and sectioned to 5 µm for histological analysis and 40 µm for analysis by second-order harmonic imaging.

Airway smooth muscle was identified by IHC staining using an alpha smooth muscle actin antibody (1:400, A5228 anti-mouse monoclonal, Merck, Taufkirchen, HE, Germany) followed by the secondary antibody Goat Anti-Mouse IgG (H+L)-HRP Conjugate (1:1000 dilution, 1721011, Bio Rad Laboratories, Hercules, CA, USA). The alpha-smooth muscle ratio was measured as previously described [32].

Haematoxylin and Eosin staining was conducted with Mayer’s Haematoxylin and Eosin (Sigma-Aldrich, St. Louis, MO, USA) as per the manufacturers’ instructions. Airway epithelial thickness in small and large airways was measured as previously described [32]. The mean linear intercept was determined as previously described [33].

The PolySciences Picrosirius Red Kit (24901A-500, Polysciences Inc., Warrington, PA, USA) was used according to the manufacturer’s instructions to stain for collagen and manually quantified using Image J (Version 1.53t) on three non-overlapping sections of red staining around the airway and then averaged to determine airway collagen. The collagen distribution was confirmed using second-order harmonic imaging as previously described [34]. Briefly, lung images were taken on a Leica STED SP8 Multi-Photon microscope, and channel separation was performed using the Leica Application Suite X software (Leica, Microsystems, Weltzar, Germany). The second-harmonic-generation signal was collected between 490 and 580 nm, and autofluorescence was collected between 500 and 800 nm. The proton pulse rate was set to 800 fs, and images were taken using a 25× objective. The images were the summed sliced of the 3D z stack, and a gamma correction of (0.7) was applied. The images were analysed using Fiji ImageJ (National Institutes of Health, Bethesda, MD, USA) to quantify the total amount of collagen. The representative images were the merger of three generated channels produced by second-order harmonics: forward (cyan), reverse (magenta), autofluorescence. Forward and reverse scatter were summed to provide the percentage of collagen for each airway.

### 2.4. Real-Time PCR

Total mRNA was extracted from the right lung lobes using TRIzol^TM^ reagent (Invitrogen, ThermoFisher, Waltham, MA, USA) as previously described [35]. Purified mRNA was used to generate the template to create first-strand cDNA using the Sensifast cDNA synthesis kit (Bioline Meridian Bioscience, London, EX, UK) according to the manufacturer’s instructions. The SensiFASTÔ SYBR Hi-ROX Kit (Bioline Meridian Bioscience, EX, UK) was used for the quantification of mRNA expression (Bio-Rad CFX96 Real-Time System) of genes of interest (Table 1). *β-actin* was used as a housekeeping gene (Table 1). mRNA expression was quantified against *β-actin* using the 2^−ΔΔCT^ method and expressed as the fold change of the sham group.

### 2.5. Statistical Methods

All data are presented as the mean ± the standard error of the mean (SEM) and analysed using one-way or two-way ANOVA followed by Tukey’s post hoc tests (GraphPad Prism 9.4.1, GraphPad, Boston, MA, USA). *p* < 0.05 was considered statistically significant.

## 3. Results

### 3.1. Third-Hand E-Vapour Exposure Has No Effects on Body or Organ Weight

To assess the effects of 4-week exposure to third-hand e-vapour, the mouse body and organ weights were assessed at the endpoint. Exposure to third-hand e-vapour with (18 mg) or without (0 mg) nicotine had no effects on body or organ weight, when compared to sham-exposed controls (Table 2).

### 3.2. Exposure to Third-Hand E-Vapour with Nicotine Reduces Bronchial Responsiveness to Provocation, but Has Limited Effects on Airway Inflammation

**Figure 1:**
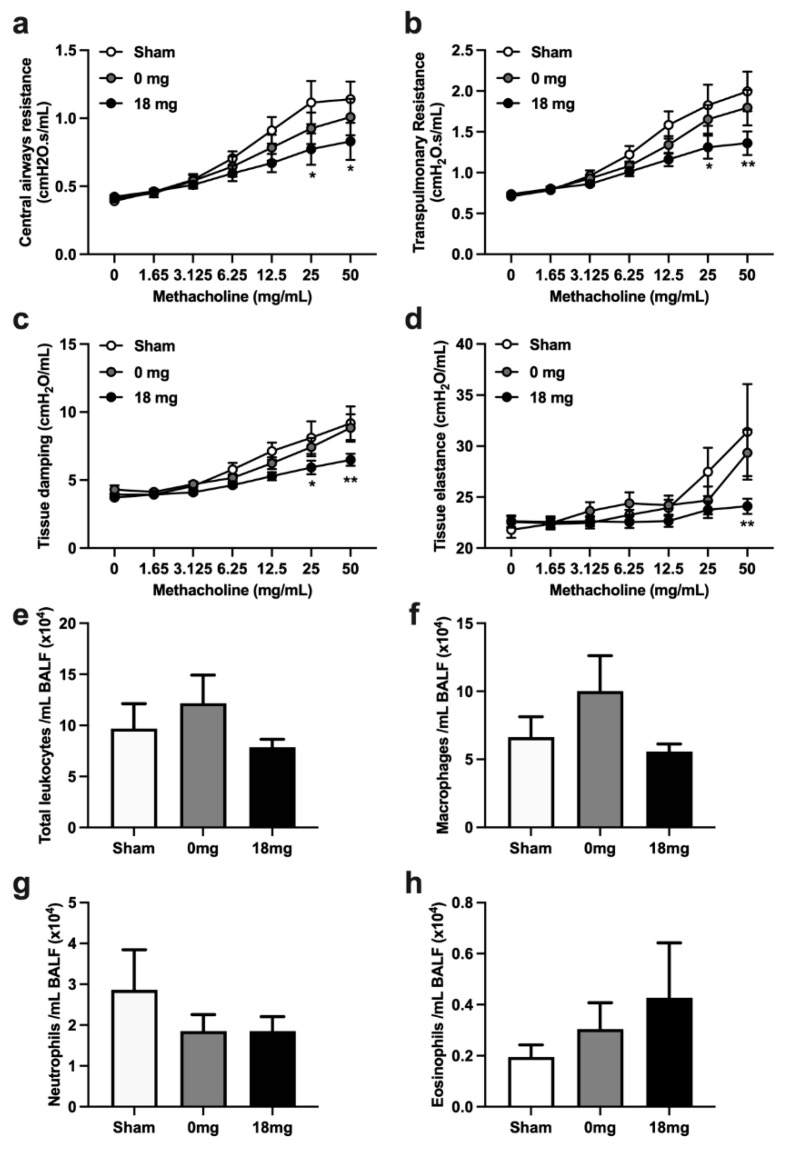
Exposure to third-hand e-vapour with nicotine reduces bronchial responsiveness to provocation, but has limited effects on airway inflammation in mice exposed to 4 weeks of third-hand e-vapour. Lung function in terms of (**a**) central airway resistance, (**b**) transpulmonary resistance, (**c**) tissue damping, and (**d**) tissue elastance, in response to increasing doses of nebulised methacholine. (**e**) Total leukocytes, (**f**) macrophages, (**g**) neutrophils, and (**h**) eosinophils in the bronchoalveolar lavage fluid. Data are expressed as the mean ± SEM and analysed by two-way ANOVA (**a**–**d**) or one-way ANOVA (**e**–**h**) with Tukey’s post hoc tests, *n* = 7–10/group. * *p* < 0.05, ** *p* < 0.01 versus sham at the same concentration of methacholine.

To assess the effects of third-hand e-vapour exposure on lung function, we measured bronchial responsiveness to methacholine provocation. Exposure to third-hand e-vapour without nicotine had no effects on central and transpulmonary resistance, tissue damping, and tissue elastance following methacholine provocation compared to sham-exposed controls (Figure 1a–e). In contrast, exposure to third-hand e-vapour with nicotine reduced central and transpulmonary resistance, tissue damping, and tissue elastance following methacholine provocation, compared to sham-exposed controls (Figure 1a–d).

Interestingly, exposure to third-hand e-vapour with or without nicotine had minimal effects on airway inflammation (Figure 1e–h); however, there were trends toward decreased neutrophil (Figure 1g) and increased eosinophil (Figure 1h) numbers compared to sham-exposed controls. Collectively, these data show that third-hand e-vapour exposure in the presence of nicotine impairs lung function and may suppress some features of airway inflammation.

### 3.3. Exposure to Third-Hand E-Vapour, with or without Nicotine, Has No Effect on Airway Smooth Muscle Actin

**Figure 2:**
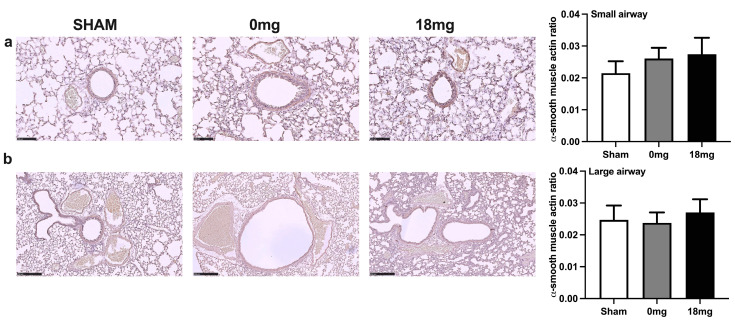
Exposure to third-hand e-vapour, with or without nicotine, has no effect on airway smooth muscle actin. Alpha smooth muscle actin staining and quantification in (**a**) small (20× magnification, a scale bar = 100 μm) and (**b**) large (10× magnification, a scale bar = 250 μm) airways of mice exposed to 4 weeks of third-hand e-vapour with (18 mg) or without (0 mg) nicotine. Data are expressed as the mean ± SEM and analysed by one-way ANOVA with Tukey’s post hoc tests, *n* = 6–9/group.

We next explored whether changes in airway smooth muscle occurred alongside the reduced bronchial responsiveness observed by performing immunohistochemical staining for alpha smooth muscle actin. Exposure to third-hand e-vapour, with or without nicotine, had no statistically significant effects on airway smooth muscle thickness in both the small (Figure 2a) and large airways (Figure 2b) compared to sham-exposed controls. However, there was a small trend towards increased small airway smooth muscle thickness following third-hand e-vapour exposure (Figure 2a).

### 3.4. Exposure to Third-Hand E-Vapour with Nicotine Induces Remodelling of the Airway Epithelium and Emphysema-Like Alveolar Enlargement

**Figure 3:**
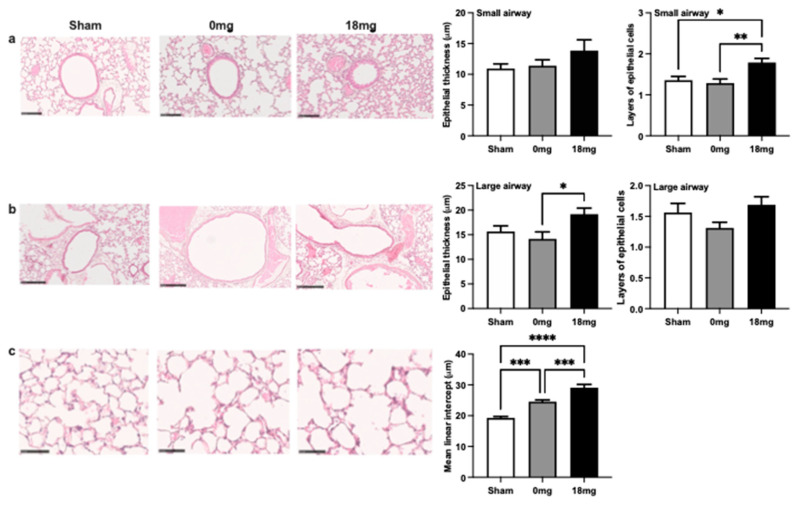
Exposure to third-hand e-vapour with nicotine induces remodelling of the airway epithelium and emphysema-like alveolar enlargement. Haematoxylin and Eosin staining and quantification in (**a**) small (20× magnification, a scale bar = 100 μm) and (**b**) large airways (10× magnification, a scale bar = 250 μm) airways of mice exposed to 4 weeks of third-hand e-vapour with (18 mg) or without (0 mg) nicotine. Representative images of parenchyma (40× magnification, a scale bar = 50 μm) and (**c**) mean linear intercept quantification Data are expressed as the mean ± SEM and analysed by one-way ANOVA with Tukey’s post hoc tests, *n* = 7–8/group. * *p* < 0.05, ** *p* < 0.01, *** *p* < 0.001, **** *p* < 0.0001.

To assess the effects of third-hand e-vapour exposure on the airway epithelium, the epithelial thickness and layers of epithelial cells were measured (Figure 3a,b). Exposure to third-hand e-vapour without nicotine had no effects on small or large airway epithelial thickness or the number of epithelial layers (Figure 3a,b). In contrast, exposure to third-hand e-vapour with nicotine specifically increased the number of epithelial layers in small airways (Figure 3a) and epithelial thickness in large airways (Figure 3b).

Interestingly, exposure to third-hand e-vapour with or without nicotine induced emphysema-like alveolar enlargement, where exposure in the presence of nicotine resulted in a more-pronounced emphysematous phenotype (Figure 3c). Together, these data demonstrate that sub-chronic exposure to third-hand e-vapour containing nicotine induces small and large epithelial remodelling and emphysema-like alveolar enlargement.

### 3.5. Exposure to Third-Hand E-Vapour with Nicotine Increases Small Airway Collagen Deposition

**Figure 4:**
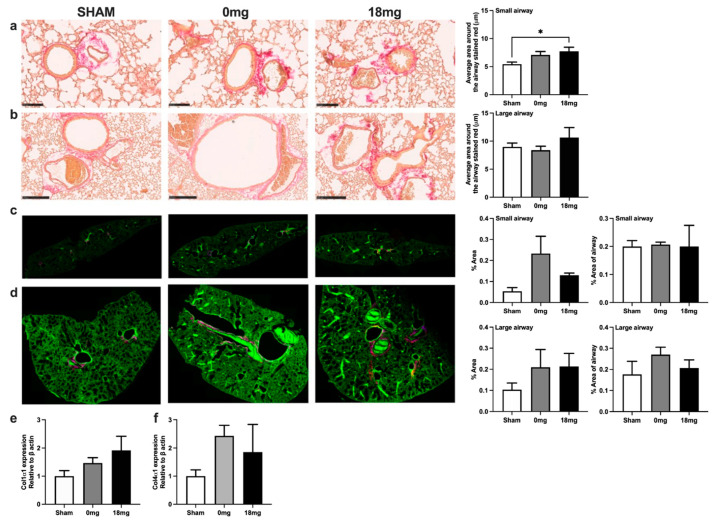
Exposure to third-hand e-vapour with nicotine increases small airway collagen deposition. Picrosirius red staining and quantification in (**a**) small (10× magnification, a scale bar = 250 μm) and (**b**) large (20× magnification, a scale bar = 100 μm) airways of mice exposed to 4 weeks of third-hand e-vapour with (18 mg) or without (0 mg) nicotine. Second-order harmonics merged representative images (25× magnification) and analyses of lung sections in small airways (**c**) and large airways (**d**). Gene expression of (**e**) collagen 1a1 and (**f**) collagen 4a1 expressed relative to B-actin. Data are expressed as the mean ± SEM and analysed by one-way ANOVA with Tukey’s post hoc tests, n = 3–8. * *p* < 0.05.

To assess the effects of third-hand e-vapour on tissue remodelling, collagen deposition was measured by picrosirius red staining (Figure 4a,b), second-order harmonics (Figure 4c,d), and qPCR (Figure 4e,f). Exposure to third-hand e-vapour with nicotine increased airway collagen in the small airways, but not large airways, compared to sham-exposed mice (Figure 4a,b). Exposure to third-hand e-vapour with or without nicotine showed a trend towards increased total collagen in the small and large airways generated by forward and reverse scatter by second-order harmonics (Figure 4c,d). Exposure to third-hand e-vapour with or without nicotine trended to increase lung *col1α1* and *col4α1* gene expression compared to sham-exposed controls (Figure 4e,f). Together, these data suggest that third-hand exposure, irrespective of the presence of nicotine, may drive collagen deposition in the lungs.

## 4. Discussion

The major finding of this study is that sub-chronic exposure to third-hand e-vapour, with or without nicotine, induces small airway collagen deposition and emphysema-like alveolar enlargement in the absence of substantial inflammation. Our study is the first to report the detrimental pulmonary effects of sub-chronic third-hand e-vapour exposure.

Using our mouse model of third-hand e-vapour exposure, we first assessed the effects of exposure on body and organ weights and showed that these were not altered after 4 weeks. There were no changes in brain weight, which differs from our 8-day acute third-hand exposure study, which showed that non-nicotine-containing third-hand e-cigarette exposure caused a significant reduction in brain weight [31]. In addition, we did not observe changes in retroperitoneal fat pad mass, which was surprising given that nicotine and propylene glycol are known to reduce body and visceral fat in mouse models [36].

Similar to our previous third-hand 8-day acute exposure model, we found that 4 weeks of exposure to nicotine-containing third-hand e-vapour robustly decreased bronchial responsiveness to methacholine provocation [31]. This is likely due to the presence of nicotine, as nicotine is a competitive agonist of methacholine (i.e., they both bind to the same receptor), and its presence may have caused bronchodilation, which reduced the contractile responses in the airways [37].

We next assessed airway inflammation and found a reduction in airway immune cells, which is similar to our previous observations in our acute model [31]. The trend to a reduction in neutrophils is consistent with other studies that have reported anti-inflammatory effects of chronic low-dose e-cigarette exposure [12,38]. However, whether third-hand e-vapour exposure induces a pro-inflammatory response remains to be fully determined, but studies assessing a time-course of third-hand e-vapour exposure, including longer chronic models, are warranted.

Third-hand e-vapour exposure, with or without nicotine, resulted in emphysema-like alveolar enlargement and remodelling. This finding is consistent with third-hand smoke and firsthand vaping studies, where exposed rodents show significant alveolar disruption in response to chronic exposure [39,40,41]. Histological analysis of both the small and large airways showed changes in lung morphology, including increases in epithelial cells within the small airway and epithelial thickness within the large airway and small airway collagen deposition, with the effects more pronounced in the small airways. These findings are consistent with firsthand e-cigarette studies that show that the small airways are more affected [11]. The airway epithelium had a hyperplastic appearance in a nicotine-dependent manner, and in the absence of any significant airway inflammation, our data suggest that nicotine-containing third-hand exposure may cause airway remodelling independent of airway inflammation.

Likewise, collagen organisation and distribution were significantly increased in small airways following exposure to nicotine-containing third-hand e-vapour. Nicotine has been shown to activate fibroblasts that upregulate the synthesis of collagen [42]. The development of fibrosis is traditionally considered to be secondary to inflammation, but we and others have demonstrated that fibrosis can occur independent of inflammation [43] and is not changed in humans with asthma following treatment with inhaled corticosteroids [44].

Previous studies have shown that, in some instances, the pathophysiological features of airway reactivity, airway inflammation, and airway remodelling can exist as distinct entities. For example, in a murine model of cigarette smoke exposure, targeted inhibition of miR-21 suppressed smoke-induced airway inflammation and small airway collagen deposition, but not emphysema-like alveolar enlargement [45]. In a different study, acute cigarette smoke exposure in mice increased airway inflammation with no effects on methacholine-induced small airway contraction [46]. In other studies, miR-21 inhibition in steroid-insensitive allergic airway disease in mice robustly suppressed steroid-insensitive airway hyperresponsiveness, but had no effect on allergic airway inflammation [47]. Interestingly, and in agreement with the findings of the current study, another study showed that small airway hyporesponsiveness is a reproducible feature of allergic airway disease that occurs in the presence of airway remodelling [48]. Collectively, these studies demonstrate distinctions between airway inflammation, remodelling, and tissue function in several different scenarios that are driven by different exposures/stimuli. Importantly, the observations described above support those of the current study that airway inflammation and the features of remodelling in the lung can exist as separate entities and with potentially different pathogenic drivers.

Our findings demonstrate that exposure to third-hand e-vapour can have detrimental effects on pulmonary structure and function. These findings also contest commonly held perceptions that e-cigarettes are a safe alternative to traditional tobacco cigarettes, which is untrue as, even at a third-hand exposure level, it induces negative biologic effects on the pulmonary system. Our study has several limitations that represent avenues for further investigation. Firstly, our study design reflects the current legislation and vaping habits that may change in the future. For example, different flavours, devices, and nicotine strengths may affect the observed data. We also exclusively studied the effects of exposure in male mice, limiting the ability to determine the sexually dimorphic effects of exposure to third-hand e-vapour. Finally, the current study determined the effects of 4 weeks of exposure; however, a longer study is required to understand the impacts of third-hand e-vapour exposures on aging and establishing the progression of chronic respiratory diseases.

## 5. Conclusions

Our study determined that third-hand e-cigarette exposure negatively impacts pulmonary health by impacting the small and large airways in mice. Both nicotine-free and nicotine-containing e-vapour induced significant changes to airway morphology and collagen deposition. These findings highlight the danger associated with third-hand exposures and the need for additional research into the effects of third-hand exposures on chronic lung diseases.

## Figures and Tables

**Table 1:** Primer sequences for rt-PCR.

Gene	Sybr-Labelled Probe Sequence
*β* *-actin*	Forward Primer: 5′-GATCTTGATCTTCATGGTGCTAGG-3′ Reverse Primer: 5′-TTGTAACCAACTGGGACCATATGG-3′
*Col1* *a* *1*	Forward Primer: 5′-CGTATCACCAAACTCAGAAG-3′ Reverse Primer: 5′-GAAGCAAAGTTTCCTCCAAG-3′
*Col4* *a* *1*	Forward Primer: 5′-TTCTCTTCTGCAACATCAAG-3′ Reverse Primer: 5′-GAATCTGAATGGTCTGACTC-3′

Col: collagen.

**Table 2:** Mouse body and weight data following 4-week third-hand e-vapour exposure.

	Sham	0 mg	18 mg
**Body Weight (g)**	22.49 ± 0.30	22.54 ± 0.21	22.49 ± 0.27
**Brain (g)**	0.311 ± 0.004	0.313 ± 0.005	0.308 ± 0.004
**Brain (%)**	1.38 ± 0.02	1.38 ± 0.03	1.371 ± 0.02
**Liver (g)**	1.267 ± 0.0366	1.194 ± 0.030	1.215 ± 0.032
**Liver (%)**	5.42 ± 0.10	5.30 ± 0.11	5.44 ± 0.13
**Kidney (g)**	0.185 ± 0.005	0.181 ± 0.004	0.181 ± 0.003
**Kidney (%)**	0.82 ± 0.01	0.80 ± 0.02	0.83 ± 0.01
**Spleen (g)**	0.099 ± 0.009	0.096 ± 0.002	0.095 ± 0.003
**Spleen (%)**	0.40 ± 0.01	0.43 ± 0.01	0.42 ± 0.01
**Retroperitoneal fat (g)**	0.102 ± 0.009	0.105 ± 0.007	0.117 ± 0.008
**Retroperitoneal fat (%)**	0.47 ± 0.03	0.47 ± 0.03	0.52 ± 0.04
**Epididymal fat (g)**	0.379 ± 0.022	0.384 ± 0.011	0.389 ± 0.020
**Epididymal fat (%)**	1.71 ± 0.04	1.77 ± 0.05	1.71 ± 0.07
**Gastrocnemius muscle (g)**	0.268 ± 0.010	0.287 ± 0.009	0.283 ± 0.005
**Gastrocnemius muscle (%)**	1.18 ± 0.03	1.27 ± 0.04	1.26 ± 0.02
**Tibialis muscle (g)**	0.097 ± 0.008	0.088 ± 0.007	0.095 ± 0.006
**Tibialis muscle (%)**	0.43 ± 0.04	0.39 ± 0.03	0.42 ± 0.03

Results are expressed as the mean ± SEM, n = 10/group. % = percentage of body weight.

## Data Availability

All datasets are available upon request. All information used in this study is publicly available and referenced in the Materials and Methods Section.
